# Medical Items

**Published:** 1896-06

**Authors:** 


Germain Sée, the distinguished French physician, died last month in Paris, aged seventy-eight.

---

Dr. W. C. Gibson, of Macon, died April 25th, while on a visit to Vineville, in the thirty-ninth year of his age.

---

Dr. W. F. Brunner, of Savannah, has been appointed by the Secretary of the Treasury Medical Officer of the Government in Cuba.

---

It is said that English will be recognized as one of the official languages at the coming meeting of the International Medical Congress at Moscow.

---

Here is another consumption cure, called *anti-microbia*. It is a compound of ozone and cod-liver-oil, and is administered hypodermically. Of ninety patients treated by this method, "all have been cured." Next!

---

Dr. Wm. A. Strother, of Albany, died May 1st, of pneumonia, aged fifty-six years. He was a Confederate veteran; entered the war as a lieutenant, was soon made surgeon, and served in this capacity till the end of the war. Dr. Strother had acquired a large practice, and his death is a great loss to his community.

---

The May issue of the *Medical Mirror* is a souvenir number of the *American Medical Association*, and reflects great credit upon the artistic taste and managerial skill of the editor and owner, that prince of good fellows and high-chief of the Chutmucks, Dr. I. N.

Love, of St. Louis. The Mirror and Dr. Love—long may they shine!

---

AT the recent meeting of the American Association of Physicians, which met at Washington two weeks ago, the following officers were elected: Dr. J. M. Da Costa, President; Dr. F. C. Shattuck, Vice-President; Dr. I. Minis Hays, Recorder; Dr. Henry Hun, Secretary; Dr. W. W. Johnston, Treasurer; Dr. I. E. Atkinson, Councilor.

---

FOLLOWING are the officers of the Alabama Medical Association for the current year: Dr. B. W. Toole, Talladega, President; Dr. J. A. Wilkinson, Flomaton, senior Vice-President; Dr. J. C. LeGrand, Anniston, junior vice-president; Dr. C. H. Franklin, Union Springs, and Dr. B. J. Baldwin, Montgomery, Censors for five years; Dr. R. L. Hill, Montgomery, Orator; Dr. G. C. Chapman, Birmingham, alternate Orator.

---

WE noted in a late issue the death of a lady in Chattanooga from chloroform. A subsequent development of the case was the arrest of the physicians on the charge of murder from criminal negligence. The physicians were well known and respected members of the Chattanooga profession. At the preliminary trial the presiding judge denounced the charges as being without character in fact, law, or morals, and discharged the defendants.

---

OUR readers will learn with sincere regret of the death of the venerable Dr. H. V. M. Miller, which occurred May 31st. For the last three or four years Dr. Miller's health has been failing, and his final and fatal illness began about two weeks ago. He was in his eighty-third year. Dr. Miller has had a public and private career which it is not permitted many men to enjoy. Successful in his profession as a practitioner and teacher of medicine, he perhaps became better known from the part which he played in Georgia's political history thirty and forty years ago. As the news of his death comes to us as we are going through the press we have not the space now for further remarks. We will present a sketch of his eventful life in our next issue. We beg to assure the relatives of Dr. Miller of our sympathy and condolence.

---

FRAUD! BEWARE!—The Henry Pharmacal Company, of Louisville, Ky., has been many times addressed in the past twelve months with reference to a so-called solution for Plafayer's Adjustable Cloth Roller Splint which some fraud unknown to them claims they manufacture. It is the method of this rascal to sell the formula for a fixed price, $5.00 or more, with a promise that the Henry Pharmacal Company will forward them a liberal quantity of the solution. Mr. Henry hereby wishes to warn the profession that he owns no such formula nor has any connection with any other interest other than those known in the advertising columns of this JOURNAL at present. The latest heard from this party was from Beaver Falls, Pa., and he seems to be traveling eastward.

---

THE Committee on Organization of the Second Pan-American Medical Congress has elected Dr. Manuel Carmona y Valle president, Dr. Rafael Lavista vice-president, and Dr. Eduardo Liceaga secretary, and has announced November 16, 17, 18, 19, 1896, as the date of the meeting to be held in the City of Mexico.

The most cordial invitation is extended to the medical profession of the United States to attend and participate in the meeting.

Titles of papers to be read should be sent at the earliest practicable date to Dr. Eduardo Liceaga, Calle de San Andres num 4, Ciudad de Mexico D. F. Republica Mexicana.

Those who contemplate attending should send their names and addresses at as early a date as possible to Dr. Charles A. L. Reed, St. Leger Place, Cincinnati, that the committee in Mexico may be advised of the probable attendance.

---

THE banquet of the Medical Editors Association at the Kimball House, Atlanta, on the evening of May 5th, brought together some well known representatives of the medical press and other prominent medical men, and a jolly occasion it was. After a delightful and exhilarating, but non-intoxicating, menu, short talks were made by Dr. George M. Gould, formerly of the *Medical News;*

Dr. J. C. Culbertson, of the *Lancet-Clinic;* Dr. J. C. LeGrand, of the *Alabama Medical and Surgical Age;* Dr. John B. Hamilton, of the *Journal of the American Medical Association;* Dr. I. N. Love, of the *Medical Mirror;* Dr. R. Beverly Cole, of California; Dr. H. O. Marcy, of Boston; Dr. Alonzo Garcelon, of Maine; Dr. L. S. McMurtry, of Louisville; Dr. C. A. L. Reed, of Cincinnati; and H. D. Didama, of New York. Among others present were Dr. H. A. Hare, of the *Therapeutical Gazette;* Dr. H. B. Ellis, of the *Southern California Practitioner;* Dr. Chas. W. Fassett, of the *Medical Herald;* Dr. T. D. Crothers, of the *Journal of Inebriety;* Dr. L. B. Grandy, of the ATLANTA MEDICAL AND SURGICAL JOURNAL; Dr. Nicholas Senn, of Chicago; Dr. Geo. M. Sternberg, U. S. Army; Dr. H. A. Kelley, of Baltimore; Dr. W. E. B. Davis, of Birmingham, and others.

---

WE noticed that several of our advertisers had good exhibits at the American Medical Association.

Messrs. Amour and Company, who of late years have been manufacturing some excellent preparations, had an attractive exhibit.

Fairchild Brothers & Foster displayed their various products, all of which could be called standard, and needing little introduction.

Ch. Marchand had his well known and useful preparations on display. They have made for themselves a well indicated usefulness in medicine and surgery.

Mellin's Food is so well known that a letter addressed "Mellin's Food, U. S. A.," would reach its destination. It has been the standard for years.

Sharp & Dohme, of New York and Baltimore, manufacture a line of tablets, etc., the requisite to the sale of which is simply S. & D. on the label.

It is needless to say that all the displays were made up with special care and attractiveness, and that they were in charge of well informed, courteous, and untiring gentlemen, who spared no trouble to keep the profession in mind of their different articles.

D. Appleton & Co., New York, and W. B. Saunders, Philadelphia, made a good display of their standard line of medical publications.

